# An interpretive review of selective sweep studies in *Bos taurus* cattle populations: identification of unique and shared selection signals across breeds

**DOI:** 10.3389/fgene.2015.00167

**Published:** 2015-05-13

**Authors:** Beatriz Gutiérrez-Gil, Juan J. Arranz, Pamela Wiener

**Affiliations:** ^1^Departamento de Producción Animal, Universidad de LeónLeón, Spain; ^2^Division of Genetics and Genomics, Roslin Institute and R(D)SVS, University of EdinburghMidlothian, UK

**Keywords:** cattle, breeds, selection signals, candidate genes, diversity, selective sweep, domestication

## Abstract

This review compiles the results of 21 genomic studies of European *Bos taurus* breeds and thus provides a general picture of the selection signatures in taurine cattle identified by genome-wide selection-mapping scans. By performing a comprehensive summary of the results reported in the literature, we compiled a list of 1049 selection sweeps described across 37 cattle breeds (17 beef breeds, 14 dairy breeds, and 6 dual-purpose breeds), and four different beef-vs.-dairy comparisons, which we subsequently grouped into core selective sweep (CSS) regions, defined as consecutive signals within 1 Mb of each other. We defined a total of 409 CSSs across the 29 bovine autosomes, 232 (57%) of which were associated with a single-breed (Single-breed CSSs), 134 CSSs (33%) were associated with a limited number of breeds (Two-to-Four-breed CSSs) and 39 CSSs (9%) were associated with five or more breeds (Multi-breed CSSs). For each CSS, we performed a candidate gene survey that identified 291 genes within the CSS intervals (from the total list of 5183 BioMart-extracted genes) linked to dairy and meat production, stature, and coat color traits. A complementary functional enrichment analysis of the CSS positional candidates highlighted other genes related to pathways underlying behavior, immune response, and reproductive traits. The Single-breed CSSs revealed an over-representation of genes related to dairy and beef production, this was further supported by over-representation of production-related pathway terms in these regions based on a functional enrichment analysis. Overall, this review provides a comparative map of the selection sweeps reported in European cattle breeds and presents for the first time a characterization of the selection sweeps that are found in individual breeds. Based on their uniqueness, these breed-specific signals could be considered as “divergence signals,” which may be useful in characterizing and protecting livestock genetic diversity.

## Introduction

The genetic diversity of livestock species is an economical and cultural inheritance from our ancestors, and an indispensable resource to meet the unpredictable needs of our future (Larson et al., 1992). The history of this diversity involves the spread of livestock populations from their centers of domestication as small samples of the original domesticated populations. Under new environments and the effects of genetic drift and natural selection, the different groups developed into distinct local populations (FAO, 2013). Associated with later advances in animal husbandry and breeding, more specialized breeds and breeding lines were developed. During the past 250 years, there has been a development of individually uniform but collectively highly diverse and distinguishable populations, which are known as “standardized breeds” (FAO, 2013).

In livestock populations, approximately half of the genetic diversity is shared across breeds while the other half is observed within single breeds (Sponenberg and Bixby, 2007). Hence, the substantial loss of biodiversity associated with the loss of a breed means that effective management of breeds is essential to managing the overall biodiversity of domesticated species.

During the establishment of modern livestock breeds, the genomes of domestic animal species have been subjected to multiple human-imposed selection events influencing traits of concern to agriculturists. In comparison to natural selection, artificial selection has the ability to rapidly change the genome. Selection not only affects the favored mutation but it produces a “hitchhiking” effect on the frequency of neutral alleles at linked loci (Maynard Smith and Haigh, 1974; Kaplan et al., 1989). Selection-mapping or hitchhiking mapping approaches exploit this phenomenon by searching for genomic regions of reduced variability as signatures of strong positive selection, with the aim of identifying causal mutations controlling selected phenotypes (e.g., Kohn et al., 2000; Harr et al., 2002; Storz et al., 2004; Pollinger et al., 2005; Voight et al., 2006). The different methods developed for detection of selection signatures through the analysis of genetic markers are based either on the distribution of allelic frequencies or the properties of haplotypes segregating within populations, or on the distribution of genetic differentiation between populations (reviewed by Hohenlohe et al., 2010).

In recent years, the availability of high-density, genome-wide single nucleotide polymorphism (SNP) arrays and parallel progress in statistical techniques have allowed the identification of genomic regions that have been subjected to positive artificial selection in livestock species (“selection scans”). While identifying a selection signature in the same region in different breeds gives support to the hypothesis that a particular genomic region has undergone selection for a given trait, many selection signatures appear to be breed-specific. By comparing the results of the studies that have searched for selection signatures in different cattle breeds, this review provides a map of selection footprints that could be considered a source of genetic diversity in these domestic populations and therefore represent a valuable resource that may be worth protecting independently of the productive ability of the breed(s) involved.

## Genetic diversity and selection signature studies in *Bos taurus* cattle

Present day cattle breeds are the result of years of human selection, adaptation to different environments and cross-breeding, as well as demographic effects such as bottlenecks and migration, all of which contribute to the current patterns of genetic diversity (Bruford et al., 2003; Laloe et al., 2010). Human-mediated selective processes include those related to domestication, breed formation, and ongoing selection to enhance performance and productivity. In 2009, the Bovine HapMap Consortium presented the first detailed genome-wide characterization of the genetic variability of 19 geographically and phylogenetically diverse bovine breeds, based on the analysis of 37,470 SNPs. This study showed that taurine breeds (*Bos taurus*) showed a lower genetic diversity than indicine breeds (*Bos indicus*), probably due to a lower diversity within the pre-domestication ancestral population and/or post-domestication effects of stronger bottlenecks at breed formation and stronger selection for docility and productivity (Bovine HapMap Consortium, 2009). The authors concluded that despite the decline in effective population size (*N*_*e*_) of some breeds, overall genetic diversity in cattle was “not low” and the between-breed differences in diversity were due to events at and before breed formation rather than differences in the intensity of natural or artificial selection post-domestication. This study was the first to perform a high-resolution, genome-wide examination of the structure of the cattle genome in different breeds and reported selection signatures in regions involving genes known to harbor causal mutations related to production traits (e.g., *GDF-8* and *ABCG2*, in relation to muscle conformation and milk composition, respectively) and genes associated with food conversion efficiency (e.g., *R3HDM1*). Since this initial analysis, many studies have followed, with the common aim of identifying specific genomic regions influenced by artificial selection in cattle breeds.

This review compiles the results of 21 genomic studies of European-related *Bos taurus* populations and thus provides a general picture of the selection signatures in taurine cattle identified by genome-wide selection-mapping scans. By performing a systematic comparison of the results reported in the literature, we have identified those regions that are found in several breeds showing the same production characteristics, and that therefore are very likely to harbor mutations with significant effects on production traits. In general, these are the regions that have already been highlighted by the different authors, as they show the highest statistical support for the presence of a positive selection signature, and because in many cases they contain genes related to the shared production characteristics that can be viewed as selection candidates. We also show that in many cases selection signatures are also shared by breeds showing different production characteristics. These may be regions of interest in relation to metabolic homeostasis or other general traits such as disease resistance and behavior. But one of the main objectives of the interpretive survey presented herein is to highlight those regions that have been reported in a single breed. In general, results of this type are not discussed in detail by the authors, and in some cases are not presented to the reader, such that a large portion of the biological information generated through these genomic studies is never interpreted. However, we hypothesize herein that these single-breed sweeps may indicate genomic sources of unique phenotypic characteristics of the target breed for which the selection signal has been detected. Although determining the phenotype associated with these single-breed sweeps may be particularly difficult, the identification and characterization of these regions as “divergence signals” may be of value as an initial step to protect, from a genomic point of view, the wealth of livestock diversity.

## General overview of the reviewed studies

As an initial attempt to perform a systematic review of the available literature on cattle selection signals, this review targets the genome-wide selective sweep scans described in *Bos taurus* breeds of European origin and mainly focuses on the interpretation of selection sweeps associated with dairy and beef production specialization. Hence, some studies have not been considered, including studies limited to specific chromosomes (e.g., Hayes et al., 2008; Prasad et al., 2008), or studies mainly addressing *Bos indicus* (Somavilla et al., 2014), African taurine cattle breeds (Gautier et al., 2009) or cross-bred cattle (Flori et al., 2012) or studies focusing on *Bos taurus-Bos indicus* comparisons (Porto-Neto et al., 2013; Utsunomiya et al., 2013a). Exceptions were four studies that included in their larger-scale analysis some *Bos indicus* and hybrid breeds (Bovine HapMap Consortium, 2009; Qanbari et al., 2011; Ramey et al., 2013; Porto-Neto et al., 2014), although we have only considered the results reported for the European *Bos taurus* breeds. Details of the 21 studies compiled in this review are provided in Table 1, including information about the breeds analyzed and their production characteristics, the statistical method(s) used for the identification of selection signatures, the SNP-chip or dataset analyzed, and other technical details such as the version of the reference genome on which the study was based.

**Table 1:** **Summary of the genome-wide selection mapping studies included in this review**.

**Study**	**Original reference genome**	**Genotyping platform^1^**	**Number of analyzed SNPs**	**Number of regions considered in this review^2^**	**Number of breeds**	**Breeds^3^**	**Production category**	**Analysis method^4^**	**Origin of data from original study**
Boitard and Rocha, 2013	UMD3.1	50K-chip	35,564	3	1	BLO	Beef	HMM-allele frequency	Table 1
Bovine HapMap Consortium, 2009	Btau4.0	AfC-10K 1536-chip	33,326	67	19	BMAS^*^, ANG, REDA, HER, NORW, HOL, LIM, CHA, BRSW, JER, GUE, PIE, ROM NDAM^*^, SHE^*^, NEL^*^, BRA^*^, GIR^*^, SGER^*^	Dairy, Beef, Dual-purpose	F_ST_, iHS	Table 1; Table S8
Druet et al., 2013	UMD3.1	HD-chip	725,293	147^a^	12	BB, DBB, HOL, JER, LIM, HER, ANG, CHA, GUE, PIE, ROM, BRSW	Dairy, Beef, Dual-purpose	HMM-average heterozygosity	Tables S1–S12
Flori et al., 2009	Btau4.0	50K-chip	42,486	6	3	HOL, NORM, MONT	Dairy	F_ST_	Table 2
Glick et al., 2012	Btau4.0	50K-chip	41,814	25	1	ISR-HOL	Dairy	REHH	Table 3
Hayes et al., 2009	UMD3.1	TM-9323	9,323	15	2	AUSHOL-vs.-AUSANG	Beef vs. Dairy	Differences in allele frequencies	Table 2
Hosokawa et al., 2012	Btau4.0	50K-chip	40,635	11	2	JAPBL-vs.-JAHOL	Beef vs. Dairy	Differences in allele frequencies	Table 2
Kemper et al., 2014	UMD3.1	HD-chip (real and imputed)	610,123	30	8	HOL, JER, ANG, CHA, HER, LIM, MUG, SHOR	Dairy, Beef	HAPH, iHS, F_ST_	Table 2, Table 6
Lee et al., 2013b	UMD3.1	WGS	15,125,420	15	1	HAN	Beef (high intramuscular fat content)	LD-ω, CLR	Table 1
Lim et al., 2013	UMD3.1	AfM-10K	4,522	2		HAN	Beef (high intramuscular fat content)	EHH-iES	Table 1
Mancini et al., 2014	Btau4.0	50K-chip	29,848	5	5	IT-BR, IT-HOL, PIE, MAR, IT-PEZZ	Dairy, Beef, Dual-purpose	F_ST_	Table 1
Pan et al., 2013	Btau4.0	50K-chip	40,130	16	1	CHI-HOL	Dairy	EHH	Table 3
Pintus et al., 2014	Btau4.0	50K-chip	42,514	53	2	PIE-vs.-ITBR	Beef vs. Dairy	F_ST_ (LOWESS regression correction)	Table 1
Porto-Neto et al., 2014	UMD3.1	HD-chip	680,000	55	9	HAN (against six European breeds, NEL, NDAM)	Beef (high intramuscular fat content)	F_ST_	Table 2
Qanbari et al., 2010	Btau4.0	50K-chip	41,398	12	1	HOL	Dairy	REHH	Table 4
Qanbari et al., 2011	Btau4.0	50K-chip	40,595	14	10	HOL, BRSW, SIM, AUS-ANG, BELR^*^, HER, MUG, SGER, SHOR, BRA^*^	Dairy, Beef, Dual-purpose	iHS, F_ST_	Table 2
Qanbari et al., 2014	Btau4.0	WGS-based imputation	Sequenced data (15,182,131 SNPs), medium density panel (39,304), high density panel (645,189).	140	1	FLE	Dual-purpose	iHS, CLR	Tables S3, S4
Ramey et al., 2013	UMD3.1	50K-chip AFFXB1P-chip-2787037	52,942 (50kchip dataset); 2,575,339 SNPs (AFFXB1P)	132	15	ANG, BRA, CHA, HAN, HER, LIM, SAL, SHOR, SIM, BRSW, FINA, HOL, JER, WAG, BRA^*^	Dairy, Beef, Dual-purpose	Extended low diversity haplotypes	Tables 2, 3, 4
Rothammer et al., 2013	UMD3.1	50K-chip	47,651	61^b^	8	GAL, BB, RED-HOL, OBRA, MUR, FRGE, FLE, BRA^*^	Beef	XP-EEH	Tables S4–S13
Stella et al., 2010	Btau4.0	HMap-data	32,689	215^c^	5	BRSW, GUE, HOL, NORW, JER	Dairy	CLL	Within-breed results^5^
Wiener et al., 2011	Btau4.0	HMap-data	31,312	30^d^	2	CHA-vs.-HOL	Beef vs. Dairy	Difference in allele frequencies	Table S2

1 High-throughput genotyping arrays used for genotyping in the different studies 50k chip, Illumina's Bovine SNP50 Genotyping BeadChip; 1536-chip, Illumina 1536 BeadArray assays; HD-chip, Illumina Bovine HD chip genotyping assay; AfC-10K, Affymetrix Custom 10K; TM-9323, 9323 SNPs (Parallele TM/Affymetrix TM); HMap-data, data from Bovine HapMap Consortium; WGS, Whole genome sequence; AfM-10K, Affymetrix MegAllele GeneChip Bovine Mapping 10K SNP array; AFFXB1P-chip-2787037, Affymetrix Axiom Genome-wide BOS 1 assay = 2,787,037 SNPs.

2 Where indicated the initial significant positions reported by the original authors were filtered in this review as described below (number of raw reported sweeps):

a From an initial list of 398 positions, only those showing an average heterozygosity < 0.003 were considered herein.

b From an initial list of 1251 breed-specific significant positions, only those with consecutive XP-EHH values (gap < 1 Mb) higher than 4 were considered herein.

c From the initial results from the within-breed analysis results kindly provided by the authors (a total of 13,077 significant positions at P < 0.01, genome-wide), we selected, for each breed, the top 10% of the positions showing a significant CLL, and then we grouped the positions in signatures regions. Following the authors' criteria, adjacent signature regions were considered “distinct” if they were separated by at least 3 consecutive windows with non-significant CLL (P < 0.01 genome-wide).

d From the initial list of 121 significant positions of top 1% MA_d values, the positions were grouped in regions of subsequent significant regions with no more than 1 Mb gaps.

3 Breed abbreviations: ANG, Angus; AUS-ANG, Australian Angus; BB, Belgian Blue; BLO, Blonde d'Aquitaine; CHA, Charolais; GAL, Galloway; HER, Hereford; HAN, Korean Hanwoo; LIM, Limousin; MAR, Marchigiana; MUG, Murray Gray; PIE, Piedmontese; REDA, Red Angus; ROM, Romagnola; SAL, Salers; SHOR, Shorthorn; WAG, Wagyu; AUSHOL-vs.-AUSANG, Australian Holstein vs. Australian Angus; CHA-vs.-HOL, Charolais vs. Holstein; JAPBL-vs.-JAHOL, Japanese Black vs. Japanese Holstein; PIE-vs.-ITBR, Piedmontese vs. Italian Brown; BRA, Braunvieh; BRSW, Brown Swiss; CHI-HOL, Chinese Holstein; FINA, Finnish Ayrshire; GUE, Guernsey; HOL, Holstein; ISR-HOL, Israeli Holstein; IT-BR, Italian Brown; IT-HOL, Italian Holstein; IT-PEZZ, Italian Pezzeta rosa; JER, Jersey; MONT, Montbéliarde; NORM, Normande; NORW, Norwegian Red; RED-HOL, Red Holstein; DBB, Dual-purpose Belgian blue; FLE, Fleckvieh; FRGE, Franken Gelbvieh; MUR, Murnau-Werdenfelser; OBRA, Original Braunvieh; SIM, Simmental.

4 Statistical methods used in the original studies reviewed in this work: CLL, Parametric composite log likelihood of the differences in allelic frequencies; CLR, Composite likelihood ratio of a model considering a section sweep as defined by Nielsen et al. (2005); EHH, Extended haplotype homozygosity; F_ST_, Population differentiation statistic; HAPH, Haplotype homozygosity measurement; HMM allele frequency, Hidden Markov model-based test, local variations in the allele frequency; iHS, Integrated haplotype homozygosity score; LD ω, LD-based omega (ω) statistics; REHH, Relative extended haplotype homozygosity; XP-EEH, Cross population extended haplotype homozygosity.

5 Boettcher and Stella, personal communication.

* Additional breeds analyzed in the original studies but not considered in this review: BMAS, Beefmaster; BELR, Belmont Red; NDAM, N'Dama; SHE, Sheko; NEL, Nelore; BRA, Brahman; GIR, Gir; SGER, Santa Gertrudis.

Depending on the number of breeds analyzed, we classify the studies as those that focus on: (i) a single breed (Qanbari et al., 2010, 2014; Glick et al., 2012; Boitard and Rocha, 2013; Lee et al., 2013b; Lim et al., 2013; Pan et al., 2013); (ii) a pair-wise comparison of closely-related populations with divergent production characteristics (mostly beef vs. dairy breeds, e.g., Hayes et al., 2009; Wiener et al., 2011; Hosokawa et al., 2012; Pintus et al., 2014) and (iii) several breeds, from three (Flori et al., 2009) to 19 breeds (Bovine HapMap Consortium, 2009), of the same or different production characteristics, and for which both across- and within-population analyses are performed. Overall, the selection sweeps considered in this review involved 37 breeds (including 17 beef breeds, 14 dairy breeds, and six dual-purpose breeds), and four different beef-vs.-dairy comparisons (Australian Holstein vs. Australian Angus, Charolais vs. Holstein, Japanese Black vs. Japanese Holstein, Piedmontese vs. Italian Brown) (Supplementary Table S1 in Supplementary Material). In addition, we have considered those selection sweeps reported for Holstein populations from specific geographic regions, such as Italian, Israeli or Chinese Holstein cattle, for Angus and Australian Angus cattle, and for Simmental and its German strain Fleckvieh, as related to “distinct” breeds in order to investigate whether there is evidence for geographical region-specific sweeps for the same breed.

The genotyping platforms used in the considered studies demonstrate the rapid development of livestock genomic tools over the last few years (Table 1). The earliest study included, that of Hayes et al. (2009), involved the analysis of 9323 SNPs genotyped by Parallele TM or Affymetrix TM and the Bovine HapMap study (2009) generated data using a custom Affymetrix 10K genotyping chip and Illumina 1536 BeadArray assays (Taylor, personal communication). Additional analyses of the original Bovine HapMap dataset were reported later by Stella et al. (2010) and Wiener et al. (2011). But most of the studies compiled in this review (10 out of 17) are based on the medium density SNP-array platform (~50K SNPs) provided by the Illumina Bovine SNP50 Genotyping BeadChip (Matukumalli et al., 2009). This SNP-array provides an initial dataset of 54,001 SNPs of which quality control filtering left between 29,848 (Mancini et al., 2014) and 47,651 (Rothammer et al., 2013) markers available for analysis in the 10 studies considered (Table 1). The studies of Druet et al. (2013) and Porto-Neto et al. (2014) involved genotyping with the Illumina BovineHD genotyping assay (>770K SNPs), which, after quality control filtering, resulted in 680,000 and 725,293 markers, respectively. In Kemper et al. (2014) the genotypes obtained with the Illumina BovineHD chip were used to perform imputation of a second dataset generated with the Illumina Bovine SNP50v2.0 (Erbe et al., 2012), yielding a total of 616,350 and 692,527 SNPs for analysis within the groups of dairy and beef breeds, respectively. Ramey et al. (2013) used the Illumina's Bovine SNP50 Genotyping BeadChip and a pre-screening assay comprising almost 2.8 million SNPs that were used as an initial marker panel in the design of the Affymetrix Axiom Genome-wide BOS 1 assay (AFFXB1P). Finally, some of the most recent studies have used data generated by large-scale sequencing. Lee et al. (2013b) analyzed more than 15 million SNPs identified through the sequencing of 12 genomes of Hanwoo cattle, whereas Qanbari et al. (2014) performed a sequence-based imputation, from a 50K SNP panel bridged by a high-density panel to the full genome sequence of Fleckvieh individuals.

The reports reviewed here have applied different but complementary statistics to detect selection signatures (Table 1). We classify the studies in the following categories: (i) studies that have estimated differences in allele frequencies by contrasting pair of breeds through F_ST_ (or related statistics) or by differences in allelic frequencies (Flori et al., 2009; Hayes et al., 2009; Wiener et al., 2011; Hosokawa et al., 2012; Mancini et al., 2014; Pintus et al., 2014; Porto-Neto et al., 2014; the across-breed results of Stella et al., 2010); (ii) studies based on extended regions of low diversity or the calculation of extended haplotype homozygosity (EHH) or variants of this statistic such as Relative Extended Haplotype Homozygosity (REHH), the long-range haplotype (LRH) test, and integrated Haplotype Homozygosity Score (iHS) (Qanbari et al., 2010; Glick et al., 2012; Lim et al., 2013; Pan et al., 2013; Ramey et al., 2013; Rothammer et al., 2013); and (iii) studies based on the allele frequency spectrum, in which regions with outlying allele frequency patterns within a single population are identified through various tests (e.g., the CLR, Composite likelihood-ratio test; CLL, parametric composite log likelihood; and HMM, Hidden Markov Model-based test) (Boitard and Rocha, 2013; Druet et al., 2013; the within-breed results of Stella et al., 2010). The studies based on F_ST_ and related statistics (category i) detect diversifying selection between breeds. Of within-breed studies, those based on differences in allele frequency patterns (category iii) have greatest power to detect completed selection (fixation of alleles) whereas the haplotype-based procedures (category ii) have greatest power to detect ongoing selection, as they explore the structure of haplotypes and essentially identify unusually long haplotypes carrying the ancestral and derived alleles (Qanbari et al., 2014). Some of the studies implement two or three different selective sweep mapping methods that fall into multiple categories (Bovine HapMap Consortium, 2009; Qanbari et al., 2011, 2014; Lee et al., 2013b; Kemper et al., 2014) (Table 1).

## Filtering criteria and comparative approach

In order to look for independent identification of the same regions and to identify those single-breed sweeps that could be uniquely associated with individual breeds, we compiled all the selection signals as reported in the different studies. For some studies reporting both regions identified in specific breeds and also across-breed analyses (Flori et al., 2009; Stella et al., 2010; Qanbari et al., 2011), we only considered the regions reported for specific breeds. The only exception to this criterion was the inclusion in our reviewed dataset of the 12 autosomal regions with extreme F_ST_ value across all populations reported by the Bovine HapMap Consortium (2009). In all cases except one, the details of the selection signatures (Start-End of the region; candidate genes included) were obtained from the original publications (tables in the main text or Supplementary Material); the only exception was the results reported for Stella et al. (2010). In this case, we compiled the genomic positions of the 13,000 significant positions (*P* < 0.001) identified for the five individual breeds (kindly provided by the authors, Boettcher and Stella, personal communication).

For four of the studies for which the original list of significant regions/positions included the results of all the positions/windows exceeding the significance threshold (Stella et al., 2010; Wiener et al., 2011; Druet et al., 2013; Rothammer et al., 2013), we applied additional filtering criteria by selecting the most significant regions or those on the top/bottom of the distribution and/or by grouping close significant positions (within 1 Mb of distance or a distance criteria previously applied by the authors) under the same sweep signals (see Table 1 for details about the additional filtering applied to these four studies).

An important issue when comparing the results of genomic studies in cattle is related to the use of different versions of the bovine genome assembly. Nine studies were based on the UMD_3.1 reference sequence, the version currently available at Ensembl (http://www.ensembl.org/Bos_taurus/Info/Index) and the UCSC browser (genome.ucsc.edu/). Eleven out of the remaining studies provided results with reference to the previous Btau_4.0 version of the assembly (currently available at http://aug2010.archive.ensembl.org/Bos_taurus/Info/Index) whereas Qanbari et al. (2014) referred to the Btau_4.6.1 version. To make the genomic positions reported by the different studies comparable across studies, we used *LiftOver* (https://genome.ucsc.edu/cgi-bin/hgLiftOver) to translate all genomic positions to the UMD_3.1 assembly. Using default parameters, we automatically obtained the correspondence between Btau4.0/Btau4.6.1 (hereafter referred to as Btau_4) and UMD_3.1 coordinates for 403 out of the 612 regions. For the 209 other Btau_4-based regions for which the *LiftOver* analysis did not yield appropriate UMD_3.1 coordinates, we performed a manual search to provide approximate UMD_3.1 genomic positions (using the closest genes to the positions flanking the selection signal in the Btau_4 region as markers to localize the region in the UMD_3.1 reference genome).

Finally, the list of all reported selection sweeps across the 21 studies, which included a total of 1049 selection sweep regions, was sorted by UMD_3.1 genomic position. With the aim of generating an interpretable set of results, the initial 1049 selection signals were subsequently grouped into core selective sweep (CSS) regions, which were defined as signals within 1 Mb of each other. This criterion was established following a detailed analysis of the regions harboring genes such as *GDF*-8, *MC1R*, and *DGAT1*, with large phenotypic effect and previously identified as being subjected to positive selection. The flanking intervals of the defined CSSs were based on the most proximal and most distal positions of the individual selection signals included in each CSS; the breeds for which individual selection signals were included in each CSS were also noted.

## Interpretative analysis of selection sweeps reported in cattle

The number of detected selective sweeps varied across the 21 studies reviewed here (Table 1). Of the 1049 selection sweeps identified, the greatest number of regions, 215 (~20%), were obtained from the filtered data of the within-breed analysis provided by Stella et al. (2010) for five specialized dairy cattle breeds. The study from which the next highest number of regions was obtained was Druet et al. (2013) (147 regions; ~14%), who studied 12 breeds with different production characteristics (dairy, beef, dual-purpose). In contrast, four studies contributed fewer than 10 selective sweeps each to the total list (16 regions in total, ~1.5% of signals all together). These were based on breeds without wide distributions, such as French dairy breeds (Flori et al., 2009), Blonde d'Aquitaine (Boitard and Rocha, 2013), Hanwoo cattle (Lim et al., 2013), and Italian breeds (Mancini et al., 2014).

**Figure 1:**
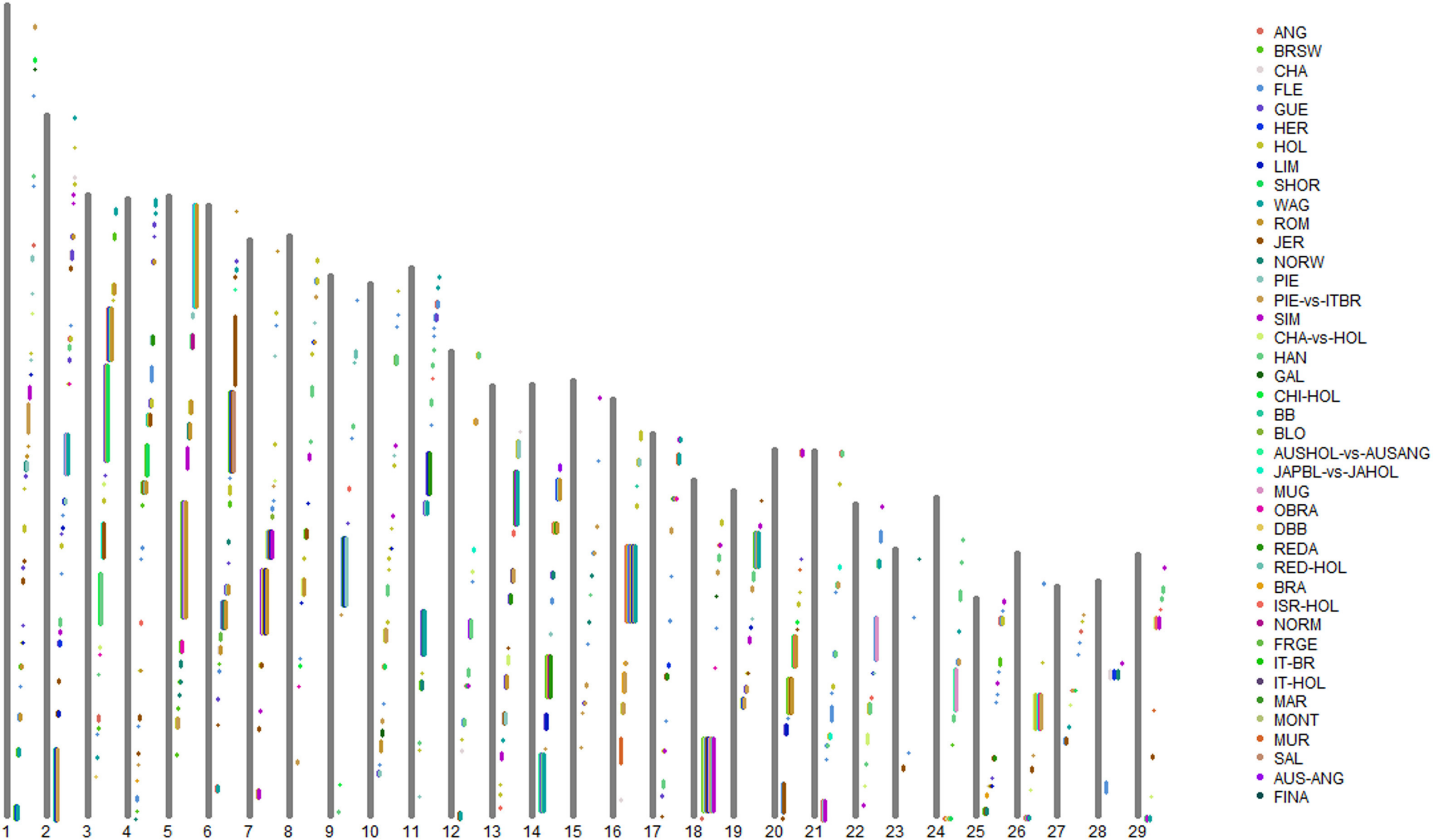
**Graphical representation of the Core Selection Sweep (CSS) regions defined in the present work across the 29 bovine autosomes, based on the selection sweeps reported by the 21 genome-wide selection-mapping scans reviewed in the present study.** Note that the proximal end of each chromosome (centromere) is represented at the bottom of the plot. ANG, Angus; AUS-ANG, Australian Angus; AUSHOL-vs.-AUSANG, Australian Holstein vs. Australian Angus; BB, Belgian Blue; BLO, Blonde d'Aquitaine; BRA, Braunvieh; BRSW, Brown Swiss; CHA, Charolais; CHA-vs.-HOL, Charolais vs. Holstein; CHI-HOL, Chinese Holstein; DBB, Dual-purpose Belgian blue; FINA, Finnish Ayrshire; FLE, Fleckvieh; FRGE, Franken Gelbvieh; GAL, Galloway; GUE, Guernsey; HER, Hereford; HOL, Holstein; ISR-HOL, Israeli Holstein; IT-BR, Italian Brown; IT-HOL, Italian Holstein; JAPBL-vs.-JAHOL, Japanese Black vs. Japanese Holstein; JER, Jersey; HAN, Korean Hanwoo; LIM, Limousin; MAR, Marchigiana; MONT, Montbéliarde; MUR, Murnau-Werdenfelser; MUG, Murray Gray; NORM, Normande; NORW, Norwegian Red; OBRA, Original Braunvieh; PIE, Piedmontese; PIE-vs.-ITBR, Piedmontese vs. Italian Brown; REDA, Red Angus; RED-HOL, Red Holstein; ROM, Romagnola; SAL, Salers; SHOR, Shorthorn; SIM, Simmental; WAG, Wagyu.

By grouping the consecutive selection sweeps reported by the different authors, (allowing gaps no greater than 1 Mb), we defined a total of 409 CSSs across the 29 bovine autosomes (Supplementary Table S2 in Supplementary Material; Figure 1), 232 (57%) of which were associated with a single-breed (Single-breed CSSs). For the remaining CSSs, we distinguished between 134 CSSs (33%) associated with a limited number (from 2 to 4) of breeds (76 two-breed CSSs, 42 three-breed CSSs, and 16 four-breed CSSs) and 39 CSSs (9.5%) that were associated with five or more breeds (from 5 to 19 breeds) (Supplementary Table S2 in Supplementary Material). We will refer to these two categories as Two-to-Four-breed CSSs and Multi-breed CSSs, respectively. In addition, four identified CSS regions were only detected in the across-breed F_ST_ analyses reported by the Bovine HapMap Consortium (2009), and will henceforth be referred to as HapMap-Unique CSSs. These four groups of CSSs are indicated by different cell color backgrounds in Supplementary Table S2, which also includes the genes that were highlighted by the original studies as possible candidate targets of the identified selection sweep.

We have also performed a thorough search of plausible candidate genes for the defined CSSs. This involved a systematic extraction of the annotated genes included in the corresponding interval of the UMD_3.1 bovine assembly using BioMart (http://www.ensembl.org/biomart/martview/). Subsequently, a systematic search for functional candidate genes was conducted by searching within CSSs for genes from four lists of genes related to phenotypes for which cattle breeds have been subjected to strong positive selection (a total of 1255 genes). These lists comprised: (i) the database of 449 genes (considering only unique genes) related to milk production and mastitis provided by Ogorevc et al. (2009); (ii) a list of 519 candidate genes for meat production and meat quality derived from the EU funded GemQual project (QLK5 – CT2000-0147; Williams et al., 2009; Sevane et al., 2013, 2014); (iii) a list of 176 genes related to coat color in cattle and other mammals (http://homepage.usask.ca/~schmutz/colors.html; Olson, 1999; Montoliu et al., 2014), and (iv) a list of 111 genes associated with stature and body size in humans and cattle (Pryce et al., 2011; Guo et al., 2012; Kemper et al., 2012) (See Supplementary Table S3 in Supplementary Material for a complete list of the candidate genes associated with the four phenotype groups). Note that some of the genes appear in more than one of the candidate gene lists (e.g., *PPARGC1A, MC1R*).

In addition, for the genes extracted for the three main CSS categories (Single-breed, Two-to-Four-breed, and Multi-breed CSSs), we carried out a functional enrichment analysis with WebGestalt (http://bioinfo.vanderbilt.edu/webgestalt/; Wang et al., 2013), using pathways defined by WikyPathways (http://www.wikipathways.org/index.php/Download_Pathways), and selecting “hsapiens_entrezgene_protein-coding” as the reference set and hypergeometric *p*-value and the “top10” options.

## Plausible candidate genes underlying CSS regions

### Overall results

The BioMart analysis extracted a total of 5182 genes from the 409 CSSs (Supplementary Table S4 in Supplementary Material). The number of genes extracted for the CSSs was proportional to the length of the genomic intervals involved in the CSSs (Table 2). Hence, a larger number of genes was extracted for the Multi-breed CSSs (2440 genes), which spanned a total of 264.05 Mb across 20 out of the 29 bovine autosomes. From the Two-to-Four-breed CSSs, which involved 202.95 Mb across all the bovine autosomes except BTA23, we extracted 1886 genes (Supplementary Table S4 in Supplementary Material).

**Table 2:** **Characterization of the four categories of core selection sweeps (CSSs) defined in this review based on the number of associated breeds**.

	**Single-breed CSS**	**Two-to-Four-breed-CSS**	**Multibreed-CSS**	**HapMap-Unique-CSS**	**Total**
Number of CSS	232	134	39	4	409
Average CSS-interval length (Mb)	73.01	202.95	264.05	1.21	541.22
Total number of extracted genes^a^	839	1886	2440	17	5182
Number of candidate genes^b^	67	83	139	2	291
Color	3	19	18	–	40
Dairy-Mastitis	36	38	54	1	129
Beef	21	19	55	1	96
Stature/Body-size	7	7	12	–	26

a Genes extracted with the web-based BioMart tool available at ensembl.org and based on the UMD_3.1 bovine genome assembly.

b Candidate genes identified through the candidate gene survey performed in this study for four phenotype classes under putative selection in modern cattle breeds.

Although more than half of the defined CSSs were associated with a single breed, and these were located across all the autosomes, the breed-specific selection sweeps spanned a shorter genomic length (73.01 Mb) and thus included a smaller number of genes (839). Seventeen of the extracted genes were located within the four regions uniquely detected in the HapMap project (Table 2). Of the 5183 genes found within CSSs, 291 of them were included in the four lists of phenotype-related candidate genes. The number of candidate genes mapping within the CSS categories defined were two (HapMap-unique CSSs), 67 (Single-breed CSSs), 83 (Two-to-Four-breed CSSs), and 139 (Multi-breed CSSs) (Table 2). The number of candidate genes for dairy, beef or body-size related traits was similar among the Single-, Two-to-Four-, and Multi-breed CSS categories, whereas coat color genes were mainly detected in the CSSs involving more than one breed (18 in the Multi-breed-CSS group and 19 in the Two-to-Four-breed category) (Table 2). Considering the three main CSS categories (Single-breed, Two-to-Four-breed, and Multi-breed CSSs), the candidate genes were not over-represented in the genes located within CSSs although the subset of dairy-related genes was slightly over-represented (but not significantly, based on Fisher's Exact Test). When the same analysis was done separately at the Single-breed CSSs and other CSSs (merging the Two-to-Four-breed and the Multi-breed CSSs together), the Single-breed category was significantly enriched (Fisher's Exact Test, *p* = 0.006) for production genes (beef and dairy) (0.07 of genes are production genes versus 0.05 for the genome overall), whereas the CSSs involving more than one breed were not significantly enriched for production genes (only 0.04 of genes are production genes).

The candidate genes highlighted by this survey are detailed in Supplementary Table S2 within the corresponding CSS where they are included. The gene symbol is indicated with different font color depending on the database of candidates from which it was identified (blue = “dairy-related,” red = beef-related,” green = “coat-color-related,” and pink = “stature/body-size-related”).

### Single-breed CSSs

The 232 single-breed CSSs identified corresponded to selection signals reported in beef (54), dairy (87) and dual-purpose (64) cattle breeds, and 28 of them were reported in a beef vs. dairy pair-wise comparison (Supplementary Table S2 in Supplementary Material). Fleckvieh showed the largest number (49) of these breed-specific selection sweeps, followed by Holstein (33 CSSs), Korean Hanwoo cattle (22), Jersey (14), Guernsey (13), and Simmental (10). Most of the Fleckvieh-specific CSSs breed were reported by Qanbari et al. (2014), and the Korean Hanwoo-related ones were reported by Porto-Neto et al. (2014). The uniqueness of these regions may be biased due to the higher marker density of these studies, which were based on whole genome sequence and the HD-chip dataset, respectively, compared with studies performed in other breeds, which were based on the lower-density SNP panels. The 33 Holstein-specific regions, however, were also extracted from studies based on lower density panels (Qanbari et al., 2010; Stella et al., 2010) and thus their abundance does not appear to be an artifact of the methodology. The large number of such CSSs may be directly related to the very strong selection and resulting high level of dairy specialization in this breed. It is not possible to present a detailed discussion of the each of the single-breed regions and associated candidate genes, but we discuss below some of the regions for which plausible candidate genes could be identified.

A number of dairy-related candidate genes were identified in Holstein-specific regions. For example, several genes related to the immune response were located in Holstein-specific regions, including *IL12B* (Subunit beta of interleukin 12), (CSS-149), which is a cytokine expressed by activated macrophages that has been found to be expressed in milk somatic cells during intra-mammary infections (Lee et al., 2006), and *TLR4* (CSS-171), for which polymorphisms have been associated with mastitis (Wang et al., 2007; de Mesquita et al., 2012) and somatic cell score in cattle (Li et al., 2014b; Wang et al., 2014a). Finally, CSS-298 includes two genes expressed in the mammary gland, *G0S2* and *LAMB3* (Ron et al., 2007). These two genes are also associated with fat metabolism in cattle (Lee et al., 2013a; Ahn et al., 2014) and therefore may be linked to the fat mobilization related to high dairy production.

For the Chinese and Israeli Holsteins, four and seven population-specific CSSs were observed, respectively. None of the Single-breed CSSs were linked to Italian Holsteins, whose selection sweeps were shared with the general Holstein population. Despite the world-wide spread of the Holstein breed, the different conditions in which the animals are reared in some of the countries e.g., resistance to heat stress in Israeli Holstein (Flamenbaum and Galon, 2010), may underlie some of these population-specific CSSs. Apart from the candidate genes suggested by Pan et al. (2013) in the Chinese study, our candidate gene survey did not detect any additional genes associated with known cattle phenotypes. Regarding this point, it should be noted that the different Holstein subpopulations shared CSSs involving major dairy candidate genes, such as the *DGAT1* (CSS-251), *ABCG2* (CSS-123), and *PLAG1* (CSS-254) genes, all of them classified as Multibreed-CSSs. For the other CSSs involving more than one breed, the Chinese Holstein was found independently of other Holstein populations in two cases included in the Two-to-Four-breed category: CSS-110 (shared with Brown Swiss, Fleckvieh, Simmental) and CSS-34 (shared with Jersey), and the Multibreed CSS-67 region (shared with Guernsey, Jersey, Korean Hanwoo, and Angus).

For two other dairy breeds, Jersey and Guernsey, several breed-specific selection sweeps were identified (14 and 13, respectively). One Guersey-related sweep (CSS-118) includes the *NFKB1* gene, whose liver expression is altered in response to pre-partum energy intake and post-partum intramammary inflammatory challenge in dairy cows (Graugnard et al., 2013). A Jersey-related sweep (CSS-383) includes *PTEN* (phosphatase and tensin homolog) which encodes a tumor suppressor gene regulating many cellular processes, including growth, adhesion, and apoptosis. *PTEN* has also recently been shown to function as an inhibitor during mammary gland development and lactation in dairy cows (Wang et al., 2014b). At the pathway level, the PTEN-AKT pathway is required for the initiation of lactation through the induction of autocrine prolactin (Chen et al., 2012). In addition, *PTEN* has been shown also to play a vital role in regulating fatty acid metabolism (Fu et al., 2012).

A number of Single-breed CSSs were identified in beef cattle breeds. There were several beef-related candidate genes located in the Angus-associated CSS-63, including *CTSK, CTSS* (cathepsin K and S), and *TMOD4* (tropomodulin 4). CTSS (cathepsin S) is known to be involved in antigen presentation and also cleaves some extracellular matrix proteins. Through its physiological role, which is to degrade type I collagen, CTSK appears to regulate adipocyte differentiation in adipose tissues of obese patients and animal models (Xiao et al., 2006; Han et al., 2009).

Beef-related candidate genes are located in several of the Korean Hanwoo-specific selection sweeps, including *ITGB3* (β3 integrin; CSS-313), which is involved in cytoskeletal organization and plays a role in the adhesion between the cell cytoskeleton and cell extracellular matrix. During postmortem aging, degradation of integrin has been found to be associated with increased drip loss in pork (Lawson, 2004), suggesting it may also be related to meat quality traits in cattle. Furthermore, *MC2R* (adrenocorticotropin receptor) and *MC5R* (melanocortin 5 receptor) are located in the Hanwoo-specific CSS-365. *MC2R* encodes a receptor for the adrenocorticotropic hormone which plays a crucial role in the regulation of glucocorticoid secretion, while *MC5R* is involved in lipid metabolism, exocrine function, and proinflammatory activity (reviewed by Switonski et al., 2013). In addition, *MC5R* expression down-regulates leptin secretion in cultured adipocytes and in humans *MC5R* polymorphisms were reported to be associated with obesity (Switonski et al., 2013). In pigs, *MC2R* is located within a QTL region for intramuscular fat content and back fat thickness (Jacobs et al., 2002) and *MC5R* is close to a QTL influencing fatness and meat quality. Several reports have confirmed an association between porcine back fat thickness or feed intake and variants of the *MC5R* gene (Emnett et al., 2001; Kováčik et al., 2012).

Several Fleckvieh-specific CSSs also include functional candidate genes. The *MFGE8* (milk fat globule-EGF factor 8 protein), located in CSS-334, has been reported to be associated with an index assessing productivity and functional and conformation traits (Fontanesi et al., 2014), which may be relevant to the dual-purpose production characteristics of this breed. The same CSS also includes *ISG20* (interferon stimulated exonuclease gene 20 kDa), which is involved in cumulus oocyte growth and may be related to fertility (Puglisi et al., 2013).

Another Fleckvieh-specific region (CSS-352) includes the *ATP2B2* gene (plasma membrane Ca(2+)-ATPase). The protein encoded by this gene is involved in the transport of calcium across the mammary cell apical membrane. This protein is related to calcium-mediated cell death and has been suggested to play a part in early signaling of mammary gland involution (Reinhardt and Lippolis, 2009).

### Two-to-Four-breed CSSs

Mapping within the Two-to-Four-breed CSS intervals, we found a high proportion of coat-color related genes (22% of the 93 candidate genes associated with these regions) including *KITLG* (*KIT*-ligand, also known as mast cell growth factor) (CSS-103; identified in Hereford, Holstein, Normande, and a Piedmontese vs. Italian Brown comparison) and *MITF* (microphthalmia-associated transcription factor) (CSS-350; identified in Fleckvieh and Murray Gray), both of which are known to be associated with coat color in cattle (Seitz et al., 1999; Hayes et al., 2010).

Several other genes that have been associated with coat color phenotypes in species other than cattle fall in Two-to-Four breed CSSs, including *HS2ST1* (CSS-68; identified in Fleckvieh, Guernsey, Japanese Black vs. Japanese Holstein, Jersey)*, AP3B1* (CSS-183; Guernsey, Piedmontese)*, MAP2K1* (CSS-185; Holstein, Piedmontese, Romagnola), *MYC* (CSS-252; identified in Holstein, Piedmontese vs. Italian Brown)*, PTS* (CSS-263; identified in Guernsey, Piedmontese vs. Italian Brown), *PDPK1* (CSS-368; identified in Brown Swiss, Jersey, Norwegian Red), and *ERCC2* (CSS-303; Angus, Simmental).

An interesting region (CSS-131) identified within the Two-to-Four-breed CSS category is that harboring the bovine casein gene cluster on BTA6 (84.66–97.99 Mb). The selection sweeps included in this CSS were identified in three dairy breeds: Braunvieh, Israeli Holstein, and Jersey. Caseins (CSN1S1, CSN1S2, CSN2, CSN3, etc.) represent the primary protein constituents of cow's milk (approximately 80%). The amount and allelic variants of caseins are associated with clotting properties and cheese yield (Wedholm et al., 2006). Due to the importance of caseins in milk production, it is intriguing that only three out of 14 dairy breeds included in this study show a selective sweep near the casein cluster. Nevertheless, this observation agrees with the discordant results reported in the 1980s and 1990s regarding the association of specific casein alleles with production traits, which appear to be breed-specific (reviewed by Caroli et al., 2009).

The *LEP* (leptin) gene appears as a strong candidate gene underlying the selection sweeps reported for one dairy (Guernsey) and two beef breeds (Piedmontese, Red Angus) (CSS-96). Leptin regulates feed intake and energy balance in mammals (Houseknecht et al., 1998) and is involved in the regulation of nutritional status and reproductive functions. Polymorphisms in the bovine *LEP* gene are associated with feed intake (Lagonigro et al., 2003) as well as production traits in both beef (Woronuk et al., 2012) and dairy cattle (Liefers et al., 2002).

### Multi-breed CSSs

Only 39 of the 409 CSSs defined herein involved at least five breeds. As was observed for the Two-to-Four-breed CSSs, the number of CSSs generally decreased as the number of breeds associated with the CSS increased. Hence, we found 12 five-breed CSSs, six six-breed CSSs, three seven-breed CSSs, three eight-breed CSS, and five nine-breed CSSs, and 10 CSSs involving 10–19 breeds.

The two CSSs involving the largest number of breeds were located on BTA6 (CSS-123) and BTA16 (CSS-278), and included 18 and 19 breeds (or pair of breeds), respectively, out of the 41 breed/breed pairs considered in this study (Supplementary Table S2 in Supplementary Material). CSS-123 involved selection sweeps reported in a large number of dairy breeds (Brown Swiss, Chinese Holstein, Guernsey, Holstein, Italian Brown, Italian Holstein, Jersey, Norwegian Red, Montbéliarde) but also beef production (Angus, Hereford, Romagnola, Piedmontese, Marchigiana) and dual-purpose (Fleckieh, MurnauWerdenfelser, OriginalBraunvieh) breeds (a selective signal was also identified for Piedmontese vs. Italian Brown, a beef-dairy comparison). This CSS includes the *ABCG2* (ATP-Binding Cassette, Sub-Family G Member 2) gene, which harbors a QTN for milk composition previously reported in cattle (Olsen et al., 2007). The precise role this gene plays in milk compositions was not initially understood but a later study suggested that *ABCG2* plays a role in mammary epithelial cell proliferation and that functional polymorphisms in this gene may influence the cellular compartment of the mammary gland and potentially milk production (Wei et al., 2012). This interval also includes the *SPP1* (osteopontin) gene, which has been shown to have significant role in the modulation of milk protein gene expression (Sheehy et al., 2009) and whose allelic variants have also been shown to be associated with variation in milk compositions (Leonard et al., 2005; Khatib et al., 2007). Possibly due to its role as a cytokine, osteopontin has been shown to be beneficial for reducing the incidence of infection during the transition period in lactating cows (Dudemaine et al., 2014). As mentioned above, CSS-123 was also identified in major beef production breeds. In this regard, the *NCAPG* gene, also located in this genomic region, harbors a causal mutation (I442M) related to fetal growth, carcass performance, and body frame size in cattle (Eberlein et al., 2009; Setoguchi et al., 2009, 2011). Interestingly, a later study has also shown a possible association of this polymorphism on milk production traits (Weikard et al., 2012). *NCAPG* overlaps with the *LCORL* (ligand dependent nuclear receptor corepressor-like) gene and in many cases these two genes are jointly referred to as *LCORL/NCAPG*. The *LCORL/NCAPG* locus influences feed intake, gain, meat and carcass traits in beef cattle (Lindholm-Perry et al., 2011) and has been associated with human height (Soranzo et al., 2009; Lango-Allen et al., 2010) and withers height in horses (Tetens et al., 2013). Another notable gene located within CSS-123 is *LAP3* (leucine aminopeptidase 3), which has been associated with milk production traits (Zheng et al., 2011). The region involving *NCAPG*, *LCORL*, and *LAP3* genes has been associated with calving ease in Norwegian Red dairy cows (Olsen et al., 2008) and in Piedmontese beef cattle (Bongiorni et al., 2012). The results in the latter breed suggest that selection on *LAP3* for better calving ease is driving the selection signature in this region. Therefore, the large number of breeds included in CSS-123 probably results from the presence of multiple genes influencing various traits of economic interest in cattle.

The region associated with 19 different breeds was CSS-278 on BTA16 (38.500–53.307 Mb). Although it involves selection sweeps reported in a large number of beef-related breeds (Angus, Australian Angus, Charolais, Hereford, Korean Hanwoo, Limousin, Piedmontese, Red Angus, Salers, Shorthorn), it was also related to dairy (Brown Swiss, Guernsey, Holstein, Jersey) and dual-purpose (Simmental, Fleckvieh, FrankenGelbvieh, Braunvieh) breeds. The BioMart extraction for this CSS interval included 253 annotated genes among which we did not identify any gene with known major effects. The genes suggested by the corresponding authors for the selection sweeps included in this CSS involve several genes related to different biological functions: immune response (*PIK3CD*, *SPSB1, ISG15, TNFRSF9*), development *(RERE*), lipid transportation (*GLTPD1)* muscle physiology (*AGRN)*, and apoptosis (programmed cell death) (*FASLG, TNFRSF1B, DFFB, TNFRSF25, DFFA, CASP9*). Among these, *CASP9* (caspase 9) is the strongest candidate as it belongs to a subgroup of proteases involved in the phase of apoptosis initiation that occurs in the post-mortem conditioning period and that, together with the calpain system, influences the ultimate meat tenderness (Ouali et al., 2006).

Our candidate gene survey in relation to CSS-278 also identified one dairy-related gene (*PEX14*, peroxisomal biogenesis factor 14), genes related to muscle physiology within the beef-candidate list (*SLC2A5*, solute carrier family 2 member 5; *TNNT2*, troponin T type 2, cardiac; *TNNI1*, troponin I type 1, skeletal, slow; *SKI*, v-ski avian sarcoma viral oncogene homolog: and *CTRC*, caldecrin), and one gene related to coat color (*ZBTB17*, Zinc Finger And BTB Domain Containing 17). *ZBTB17* is required for hair follicle structure and hair morphogenesis, and mutations in the murine gene are associated with darkened coat, dark skin, dark dermis around hairs, and abnormal follicles.

Two Multi-breed CSSs regions on BTA14 were identified in 13 and 14 breeds. One of these regions was located on the proximal end of the chromosome (CSS-251, 1.657–12.713 Mb) and involved both dairy and beef breeds (Angus, Australian Holstein vs. Australian Angus, Charolais, Charolais vs. Holstein, Chinese Holstein, Guernsey, HapMap project, Hereford, Holstein, Jersey, Korean Hanwoo, Limousin, Norwegian Red, Piedmontese, Wagyu). It is highly likely that CSS-251 incorporates the selection sweep reported in relation to the *DGAT1* (diacylglycerol O-acyltransferase 1) gene for many dairy cattle breeds, based on the causal role of the mutation K232A on milk composition (Grisart et al., 2002). In addition, the *DGAT1* gene has also been associated with carcass and meat quality traits in beef cattle (Thaller et al., 2003; Wu et al., 2012; Avilés et al., 2013). However, the *DGAT1* gene is located at the very proximal end of the chromosome (1.795–1.805 Mb), indicating that the large CSS-251 interval incorporates selection sweeps related to other genes, such as that reported near the *TG* (thyroglobulin; located at 9.262–9.509 Mb) gene. *TG* is known to influence carcass and meat quality traits in beef cattle (Gan et al., 2008; Bennett et al., 2013). Another gene highlighted by our candidate gene query in this region is *CYP11B1* (Cytochrome P450, Family 11, Subfamily B, Polypeptide 1), which influences energy metabolism. A study in German Holstein cattle has shown that SNPs in this gene are associated with milk production traits and somatic cell score independently of the *DGAT1* genotype (Kaupe et al., 2007).

The other CSS on BTA14 associated with a large number of breeds was CSS-254 (23.885–31.847 Mb). The related breeds included both dairy (Brown Swiss, Chinese Holstein, Holstein, Jersey, Montbéliarde, Normande, Norwegian Red) and beef (Angus, Braunvieh, Charolais, Limousin, Piedmontese, Red Angus) breeds as well as the dual-purpose Fleckvieh. Within this region we found the *PLAG1* (pleiomorphic adenoma gene 1) gene, which has been shown to be associated with stature in Jersey × Holstein crosses (Karim et al., 2011) but also shows pleiotropic effects on fertility such that the *PLAG1* allele associated with increased height and weight was also associated with reduced fat, greater feed intake, less residual feed intake, later puberty in both sexes, and longer post-partum interval before reconceiving in cows (Fortes et al., 2013). This region also encompasses a cluster of genes, including *CHCHD7* (coiled-coil-helix-coiled-coil-helix domain containing 7), *SDR16C5* (short chain dehydrogenase/reductase family 16C, member 5), *MOS* (v-mos Moloney murine sarcoma viral oncogene homolog), *LYN* (v-yes-1 Yamaguchi sarcoma viral related oncogene homolog), *PENK* (proenkephalin), and *RPS20* (ribosomal protein S20), that have been associated with stature in cattle and humans (Utsunomiya et al., 2013b). In particular, a polymorphism ablating a polyadenylation signal of *RPS20* has been proposed as the candidate causal mutation of a QTL influencing calving ease and stillbirth incidence in the Fleckvieh breed (Pausch et al., 2011). Another possible candidate for that CSS is *NCOA2* (nuclear receptor coactivator 2), which encodes a transcriptional coactivator for steroid receptors and nuclear receptor and has been found to influence puberty in tropical breeds of beef cattle (Fortes et al., 2011).

Also on BTA6, CSS-130 (67.850–83.375 Mb) involved selection signatures identified in 13 different breeds, involving dairy, beef, and dual-purpose cattle breeds. This region includes a cluster of tyrosine kinase receptor genes (*PDGFRA, KIT*, and *KDR*). The *KIT* (the Hardy-Zuckerman 4 feline sarcoma viral oncogene homolog) gene, which is centered in the CSS interval (71.796–71.917 Mb), explains a considerable proportion of the variation in patterned pigmentation (Hayes et al., 2010), such as the characteristic spotting phenotype of Holstein and other dairy breeds. Close to the *KIT* gene, at 71.374–71.421 Mb, *PDGFRA* (platelet-derived growth factor alpha receptor), has recently been identified as the strongest positional candidate for the non-*MC1R*-related reddening phenotype in an F2 Nellore-Angus population (Hanna et al., 2014). Other coat color genes were also located in Multi-breed CSSs. CSS-297, which contains the *MC1R* gene, was identified in 15 different breeds. Polymorphisms in this gene are related to the production of eumelanin and phaeomelanin pigments and determine the red-black axis in cattle coat color (Robbins et al., 1993). In addition, *MC1R*, through a competitive relationship for alpha-melanocyte stimulating hormone (α-MSH) with the Melanocortin 4 Receptor (appetite suppressing receptor), has been associated with growth and carcass traits (McLean and Schmutz, 2009). Some of the selection signals of the large CSS-297 interval are likely to be related to other genes. A large list of genes has been suggested by the corresponding authors, and our candidate gene survey also highlighted candidates for dairy traits (*SLC7A5*, solute carrier family 7 member 5), meat production (*CTRB1*; chymotrypsinogen B1; *FOXC2*, forkhead box C2; *CDH15*, cadherin 15, type 1, M-cadherin), and stature (*GALNS*; galactosamine (N-acetyl)-6-sulfate sulfatase).

Another coat color gene, *SILV* (*silver*), also known as *PMEL* (premelanosome protein), is located within CSS-109, related to 11 breeds (breed pairs). This gene has been associated with the white coat color characteristic of the Charolais breed (Gutiérrez-Gil et al., 2007; Kuehn and Weikard, 2007a) and pale Highland cattle (Schmutz and Dreger, 2013). It also may be involved in the gray coat phenotype of the Murray Gray breed. In addition, it has been suggested that this gene, based on its multiple splice variants expressed in a variety of tissues independent of pigmentation, could have functions other than melanosome development (Kuehn and Weikard, 2007b). The proximal section of CSS-109 also includes the *IFNG* (interferon, gamma) gene, of interest due to its relationship with the immune response.

The CSS-248 region, which was identified in seven breeds including beef breeds (Korean Hanwoo, Marchigiana, Piedmontese, Shorthorn, Simmental, Wagyu), one dairy breed (Holstein) and a beef vs. dairy comparison (Japanese Black vs. Japanese Holstein), includes the *ASIP* (agouti signaling protein) gene. Although this is a color-related gene in many species, mutations in the *ASIP* coding region have not been found to play an important role in coat color variation in cattle (Royo et al., 2005). However, a transcript variant of *ASIP* has been assumed to be the causal variant for the brindle coat color of Normande cattle (Girardot et al., 2006) and due to the expression of this gene in adipocytes and its implication in the obese yellow mouse, this transcript has also been suggested to be related to the milk composition traits in this dairy breed (Girardot et al., 2006) and intramuscular fat content in other breeds (Albrecht et al., 2012).

In addition to coat color and patterning, the presence or absence of horns is a breed hallmark in European *B. taurus*. The locus controlling the polled phenotype, *POLLED*, is located within CSS-1, on BTA1 (0.198–2.60 Mb) (Brenneman et al., 1996), which was identified in 10 breeds. The molecular basis of this phenotype has proven to be complex and the existence of allelic heterogeneity has been suggested for this locus, with the candidate causal mutations located outside known genes or regulatory regions (Drögemüller et al., 2005; Medugorac et al., 2012; Allais-Bonnet et al., 2013). Recently, a long intergenic non-coding RNA has been suggested as the most probable cause of horn bud agenesis for one of the defined allelic variants (Allais-Bonnet et al., 2013).

Another Multi-breed CSS including a gene with a major effect on a bovine qualitative phenotype is CSS-32, located at the proximal end of BTA2, and including selection sweeps described in Belgian Blue, Blonde d'Aquitaine, Limousin, Piedmontese, and a Piedmontese vs. Italian Brown breed-comparison. These are all breeds known to show disruptive or missense mutations in the myostatin (*GDF8* or *MSTN*) gene, associated with muscle conformation and in extreme cases, “double muscling” (Grobet et al., 1997; McPherron and Lee, 1997; Smith et al., 2000; Boitard and Rocha, 2013). CSS-32 encompasses the myostatin gene (2: 6.213–6.220 Mb) but also extends over a large region of BTA2 (0–13.850 Mb), due to the long selective sweep reported by Kemper et al. (2014) for Limousin, known to show very high frequency (~94.2%) of the *GDF8*-F94L mutation (Vankan et al., 2010), whereas for the other breeds, the selective sweep was closer to the *GDF*-8 location. As a result of the large size of this CSS, selective sweeps that originally did not include the *GDF*-8 gene have been incorporated under the CSS-32 label; these include two sweeps described in a Piedmontese vs. Italian Brown comparison (Pintus et al., 2014) 6.717–9.760 Mb, including the *SLC40A1* (solute carrier family 40, member 1), *COL5A2* (collagen, type V, alpha 2), *COL3A1* (collagen, type III, alpha 1), *CALCRL* (calcitonin receptor-like) and *ITGAV* (integrin, alpha V) genes, and a Fleckvieh selective sweep located in a gene-desert region. Polymorphisms in the *SLC40A1* gene have been related to beef iron content (Duan et al., 2012).

Other Multi-breed regions include genes associated with production traits. These include the short CSS-124 region, which was identified in eight breeds and includes the *PPARGC1A* (peroxysome proliferator-activated receptor-γ coactivator-1α) gene, which mediates expression of genes involved in oxidative metabolism, adipogenesis, and gluconeogenesis (Puigserver and Spiegelman, 2003). Expression of this gene has been suggested to be required for the initiation and development of lactation in dairy cattle (Weikard et al., 2005). *PPARGC1A* has also been shown to be associated with milk composition (Weikard et al., 2005; Khatib et al., 2007; Schennink et al., 2009), reproduction (Komisarek and Walendowska, 2012), growth (Li et al., 2014a), carcass traits (Shin and Chung, 2013; Ramayo-Caldas et al., 2014), and meat quality (Sevane et al., 2013).

CSS-72 (identified in eight breeds) includes *LEPR (leptin receptor)*, which due to its interaction with leptin, may be a target of selection in relation to a wide range of economically relevant traits, including growth (Guo et al., 2008), milk production (Suchocki et al., 2010), and calving interval (Trakovická et al., 2013). Finally, CSS-314 (identified in six breeds) includes the *FASN* gene, which has been associated with milk and beef fatty acid composition (Roy et al., 2006; Morris et al., 2007; Zhang et al., 2008). CSS-322 (identified in five breeds) includes *GHR* (growth hormone receptor), which has been shown to harbor a causal mutation of a QTL influencing milk yield and composition (Blott et al., 2003) and *FST* (follistatin), which encodes a protein related to ovary function and has also been suggested to play a key role in regulating bovine mammary branching morphogenesis and epithelial differentiation (Bloise et al., 2010).

We acknowledge that our candidate gene survey did not take into account genes related to the immune response and behavior, which are found in various CSSs, as reported by many of the original studies reviewed here. For example, the list of Biomart-extracted genes from all the CSS defined in this study (Supplementary Table S4 in Supplementary Material) includes genes directly associated with the immune response. Hence, the list includes 34 genes encoding proteins related to interferon and interleukin responses (16, 12, and five of them belonging to the Multi-breed, Two-to-Four-breed, and Single-breed CSSs, respectively). The CSSs also include 128 genes encoding olfactory receptors (106 of them within Multi-breed CSSs, and 11 in each of the two other categories) and 28 encoding olfactory receptor-like proteins (22 in the Multi-breed CSSs, five in the Single-breed category and one in the Two-to-Four CSSs), which are proposed to be associated with behavioral traits modified through domestication in cattle (Ramey et al., 2013) and other livestock species (Bovine HapMap Consortium, 2009).

Although our survey and the original papers have identified clear candidates for some of the Multibreed CSS regions (mainly genes influencing morphological traits but also some genes with large effects on production traits), it is worth noting that due to our method of merging multiple selection signals at similar positions under the same label, some of these CSSs involve a much larger region than that directly related to the gene with the major gene effect (as discussed above regarding the myostatin region).

## Enrichment analysis

In an attempt to highlight genes influencing traits other than those considered in our candidate gene survey and to identify the functional biological pathways that are over-represented in the genes included in the CSSs, we performed a complementary functional enrichment analysis for the genes extracted from the Single-breed, Two-to-Four-breed and Multi-breed CSS regions (Supplementary Table S5 in Supplementary Material).

Among the top 10 significant pathway terms in the Single-breed CSSs, five terms were related to the immune response [regulation of Toll-like receptor signaling, IL-1 and IL-4 signaling, and leucocyte-related validated miRNA (defined by tarBase database) pathways], and the others were linked to global metabolism (leptin signaling pathway), bone and muscle physiology (RNAKL-RANK signaling, osteopontin signaling pathways) and to one of the most important intracellular signal transduction pathways (MAPK signaling pathway).

To further explore these results and assess whether single-breed CSSs are linked to genes underlying the physiology of the production specialization for which they have been selected, we performed the functional enrichment analysis of the Single-breed CSSs, separately for the beef and dairy breeds and also for the dual-purpose breeds (Table 3). Whereas pathway terms related to the general immune response were found at similar proportions within the 10 top terms of the three subcategories (although Toll-like receptor signaling pathways terms were only identified in the analysis of the dairy CSSs), other pathway terms appeared to be subcategory-specific. For example, bone and muscle physiology-related terms constituted the majority (5/10) of the top 10 significant terms for beef breed CSSs (i.e., osteopontin signaling, RANKL-RANK signaling pathway, endochondral ossification, osteoclast signaling, striated muscle contraction) whereas those related to major metabolic pathways (leptin signaling pathway, vitamin D synthesis, insulin signaling) were found within the top 10 significant terms only in the dairy-related CSSs. For the Single-breed CSSs associated with dual-purpose breeds, the top 10 significant terms were mainly related to cell signaling pathways involving two important mitogen-activated protein kinases (*MAP2K2, MAPK3*), which are linked to pathways involving receptors of serotonin and histamine (see Table 3). Whereas serotonin is a local regulator in the mammary gland that regulates lactation and initiates the transition into the earliest phases of the involution process related to the return of the mammary gland to morphologically near pre-pregnant state (Horseman and Collier, 2014), the histamine receptors may, in addition to their involvement in local immune responses, also show central effects on modulation of behavior related to the biological function of histamine as a neurotransmitter in the central nervous system (Schneider et al., 2014). The analysis of the dual-purpose-related Single-breed CSSs also revealed over-representation of genes involved in myometrial relaxation and contraction pathways, which could be related to the selection of females that are good dairy cows and can also give birth to calves with meat-production characteristics (e.g., large size).

**Table 3:** **Results from the gene enrichment analysis performed using WikiPathway analysis (WebGestalt software; Wang et al., 2013) individually for the three production-based subcategories (beef, dairy, dual-purpose) of the Single-breed core selective sweep (CSSs)**.

**Core selective sweep (CSS) subcategory (number of genes extracted with BioMart)**	**Pathway name**	**Number of genes**	**Gene names**	**Statistics^a^**
Single-breed-CSSs involving BEEF breeds (304 genes)	Interleukin-11 signaling pathway	3	*TGFB1*, *RELA*, *IKBKB*	C = 49; O = 3; E = 0.30; *R* = 9.93; rawP = 0.0035; adjP = 0.0540
	Osteopontin signaling	2	*RELA*, *IKBKB*	C = 18; O = 2; E = 0.11; R = 18.01; rawP = 0.0054; adjP = 0.0540
	RANKL-RANK signaling pathway	3	*CTSK*, *RELA*, *IKBKB*	C = 66; O = 3; E = 0.41; R = 7.37; rawP = 0.0080; adjP = 0.0540
	Corticotropin-releasing hormone	4	*TGFB1*, *FOSL1*, *RELA*, *NCOA2*	C = 123; O = 4; E = 0.76; R = 5.27; rawP = 0.0072; adjP = 0.0540
	Endochondral ossification	3	*TGFB1*, *PTCH1*, *PLAT*	C = 69; O = 3; E = 0.42; R = 7.08; rawP = 0.0089; adjP = 0.0534
	Osteoclast signaling	2	*CTSK*, *ITGB3*	C = 19; O = 2; E = 0.12; R = 17.07; rawP = 0.0060; adjP = 0.0540
	B Cell receptor signaling pathway	4	*PIP5K1A*, *GSK3A*, *RELA*, *IKBKB*	C = 114; O = 4; E = 0.70; R = 5.69; rawP = 0.0056; adjP = 0.0540
	NOD pathway	3	*RELA, IKBKB, NLRC4*	C = 39; O = 3; E = 0.24; R = 12.47; rawP = 0.0018; adjP = 0.0540
	Androgen receptor signaling pathway	4	*KAT5, RELA, KAT7, NCOA2*	C = 91; O = 4; E = 0.56; R = 7.13; rawP = 0.0025; adjP = 0.0540
	Striated muscle contraction	2	*MYL4, ACTN3*	C = 38; O = 2; E = 0.23; R = 8.53; rawP = 0.0230; adjP = 0.0690
Single-breed-CSSs involving DAIRY breeds (239 genes)	Regulation of toll-like receptor signaling pathway	6	*MAPK13, TLR4, MAPK14, PELI2, IL12B, NFKB1*	C = 154; O = 6; E = 0.69; R = 8.75; rawP = 7.00e-05;adjP = 0.0033
	Estrogen signaling pathway	3	*ESR1, MAPK14, NFKB1*	C = 30; O = 3; E = 0.13; R = 22.46; rawP = 0.0003; adjP = 0.0047
	Toll-like receptor signaling pathway	5	*MAPK13, TLR4, MAPK14, IL12B, NFKB1*	C = 116; O = 5; E = 0.52; R = 9.68; rawP = 0.0002; adjP = 0.0047
	Leptin signaling pathway	4	*ESR1, PDE3B, MAPK14, NFKB1*	C = 81; O = 4; E = 0.36; R = 11.09; rawP = 0.0005; adjP = 0.0059
	Vitamin D synthesis	2	*CYP27A1, CYP2R1*	C = 10; O = 2; E = 0.04; R = 44.92; rawP = 0.0009; adjP = 0.0070
	TFs Regulate miRNAs related to cardiac hypertrophy	2	*RASGRF1, NFKB1*	C = 10; O = 2; E = 0.04; R = 44.92; rawP = 0.0009; adjP = 0.0070
	Focal Adhesion	5	*COL6A2, SHC3, PTEN, LAMA5, LAMB3*	C = 185; O = 5; E = 0.82; R = 6.07; rawP = 0.0015; adjP = 0.0088
	Alpha 6 Beta 4 signaling pathway	3	*MAPK14, LAMA5, LAMB3*	C = 50; O = 3; E = 0.22; R = 13.48; rawP = 0.0015; adjP = 0.0088
	Complement activation, classical pathway	2	*C1QA, C1QC*	C = 17; O = 2; E = 0.08; R = 26.43; rawP = 0.0026; adjP = 0.0136
	Insulin signaling	4	*MAPK13, SHC3, PTEN, MAPK14*	C = 163; O = 4; E = 0.73; R = 5.51; rawP = 0.0062; adjP = 0.0291
Single-breed-CSSs involving DUAL-PURPOSE breeds (195 genes)	Serotonin receptor 4-6-7 and NR3C Signaling	3	*HTR4, MAP2K2, MAPK3*	C = 18; O = 3; E = 0.06; R = 46.67; rawP = 3.50e-05; adjP = 0.0014
	Calcium regulation in the cardiac cell	5	*RGS18, RGS2, RGS1, ATP2B2, PRKAR1B*	C = 151; O = 5; E = 0.54; R = 9.27; rawP = 0.0002; adjP = 0.0041
	Serotonin Receptor 2 and ELK-SRF-GATA4 signaling	2	*MAP2K2, MAPK3*	C = 16; O = 2; E = 0.06; R = 35.01; rawP = 0.0015; adjP = 0.0205
	IL-7 signaling pathway	2	*MAP2K2, MAPK3*	C = 25; O = 2; E = 0.09; R = 22.40; rawP = 0.0036; adjP = 0.0246
	IL-9 signaling pathway	2	*MAP2K2, MAPK3*	C = 25; O = 2; E = 0.09; R = 22.40; rawP = 0.0036; adjP = 0.0246
	Myometrial relaxation and contraction pathways	4	*RGS18, RGS2, RGS1, PRKAR1B*	C = 162; O = 4; E = 0.58; R = 6.91; rawP = 0.0028; adjP = 0.0246
	Monoamine GPCRs	2	*HTR4, HRH1*	C = 33; O = 2; E = 0.12; R = 16.97; rawP = 0.0062; adjP = 0.0261
	Serotonin HTR1 group and FOS pathway	2	*MAP2K2, MAPK3*	C = 33; O = 2; E = 0.12; R = 16.97; rawP = 0.0062; adjP = 0.0261
	Hypothetical network for drug addiction	2	*MAP2K2, MAPK3*	C = 35; O = 2; E = 0.12; R = 16.00; rawP = 0.0070; adjP = 0.0261
	EPO receptor signaling	2	*MAP2K2, MAPK3*	C = 35; O = 2; E = 0.12; R = 16.00; rawP = 0.0070; adjP = 0.0261

a Wikipathway analysis Statistics: C, the number of reference genes in the category; O, the number of genes in the gene set and also in the category; E, the expected number in the category; R, ratio of enrichment; rawP, p-value from hypergeometric test; adjP, p-value adjusted by the multiple test adjustment.

In the Two-to-Four-Breed CSSs the functional gene enrichment analysis (Supplementary Table S5 in Supplementary Material) highlighted three pathway terms related to global metabolism (insulin signaling, glucuronidation, and metapathway biotransformation; the latter term involves several enzymes from the cytochrome P450 superfamily of enzymes, sulfotransferases, and glucuronosyltransferases) and others were related to the immune response [regulation of the Toll-like receptor signaling pathway and lymphocyte-validated miRNAs (TarBase)], cell adhesion mechanisms (integrin-mediated cell adhesion, focal adhesion), and specific cell physiology pathways [MAPK signaling, epithelium-related validated miRNA (TarBase), and microRNAs in cardiomyocyte hypertrophy]. The enrichment analysis performed for the Multi-breed CSSs highlighted among the top 10 significant terms, two related to the immune response (complement activation, classical pathway, complement and coagulation cascades), two related to overall lipid-metabolism (adipogenesis, SREBF, and miR33 in cholesterol and lipid homeostasis), and others related to skeleton and reproductive physiology (regulation of actin cytoskeleton, and myometrial relaxation and contraction pathways).

Because of the large number of genes highlighted by this functional analysis, we do not present here a detailed discussion about the known effects of these genes in cattle. This could be the objective of future studies focusing on some of the CSS regions presented here.

## Overall conclusions

Compilation of the results from many selection sweep mapping studies in cattle provides an ideal opportunity to investigate how artificial selection has influenced the variability and architecture of the bovine genome. Selection is likely to have eroded the levels of genetic variation that existed in the original domesticated population. At the same time, selection on a livestock breed has tended to fix specific variants that have become distinctive genetic signals of that breed compared with others. Strong selection for improvement of productivity, such as milk or beef production traits, has led to specialization of cattle breeds. It might be expected that breeds that share the same production characteristics would show a similar picture of selection sweeps related to such specialization, and conversely, that divergently specialized breeds would share few selection sweeps. However, our review shows that in many cases selection signatures are also shared by breeds showing different production characteristics. These may include regions containing genes associated with metabolic homeostasis or other general traits such as disease resistance and behavior, but may also reflect the pleiotropic effects of genes on traits relevant to both beef and dairy production. Because of the large number of selective sweeps compared here, we have not performed a detailed analysis of all genes included within the CSSs, although in a number of cases, it was possible to speculate as to which gene or genes could be the targets of selection.

This review presents an initial comparative map of the selection sweeps reported in European *Bos taurus* cattle breeds and provides an integrated dataset that can also incorporate results from future studies and thus allow the researchers to perform systematic comparisons of selection sweeps reported in cattle. This type of comparative tool is essential to properly interpret the results of individual studies for such a complex topic as selection sweeps across different breeds of the same species.

Considering the three main CSS categories defined here, the Single-breed and the Two-to-Four-breed CSS groups together accounted for about 90% of the CSSs, whereas only 9.5% of the CSSs were identified in five or more breeds (Figure 1). These Multi-breed CSSs appear to encompass the sweeps involving the limited number of genes that have large phenotypic effects across different breeds and also, in part due to the long Multi-breed CSS intervals resulting from our CSS-labeling approach, other putatively selected genes with small effect sizes, some of which are breed-specific. Regarding the large phenotypic effects linked to the Multi-breed CSSs, many of them appear to relate to physical rather than production traits, consistent with a simpler genetic architecture (i.e., fewer genes involved in determination of the phenotype) for the former. The putatively strongly selected phenotypes include physical hallmarks that define a breed, such as coat color and patterning (*MC1R*, *KIT*) or obvious morphological traits such as lack of horns (*POLL* locus) and stature (*PLAG1*). The strong signals of selection in relation to morphological traits (e.g., body size and color-patterning traits) are consistent with the theory of the “domestication syndrome” in mammals, which suggests that selective pressure for tameness during the initial stages of domestication involved a developmental reduction in neural crest cell populations and led to multiple phenotypic changes shared by various domesticated animals species (e.g., depigmentation, floppy and reduced ears, shorter muzzles, docility, smaller brain, or cranial capacity) (Wilkins et al., 2014).

In addition to the Multi-breed CSS regions including genes that influence physical traits, there are also several genomic regions that show evidence of selection across many breeds and appear to be driven by selection on production-related genes such as *ABCG2, DGAT1, NCAPG*, and *GHR.* For the CSSs including genes with large effects, there was a correspondence between the production profiles of the breeds associated with these CSSs and the known effects of the putative target gene. It is interesting that some of these genes for which the initial major effect was related to a specific specialization (e.g., *DGAT1* for milk and *NCAPG* for growth traits), latter studies have shown that they also have effects on traits of interest in the alternative production group (e.g., *DGAT1* for beef composition and *NCAPG* for milk traits). These observations for genes with known major effects provide insights into the complexity of the relationship between genes and phenotypes; this complexity may be even more pronounced for genes of small effect.

In addition to these genes with major effect, the Multi-breed CSS intervals also included other potential selection candidates related to production (dairy and beef) traits (Supplementary Table S2 in Supplementary Material), which could represent some of the small size effect genes underlying the complex genetic architecture of quantitative traits. The functional enrichment analysis for these genomic regions suggested that genes related to the immune response and reproduction traits may also have been selection targets shared by many breeds. We also found a significant over-representation of genes related to olfactory receptors (protein coding and pseudogenes) in the Multi-breed CSSs. The abundance of these genes within selection sweep intervals, which has previously been highlighted (Bovine HapMap Consortium, 2009; Ramey et al., 2013; Qanbari et al., 2014), suggests that these behavior-related loci may have played a role in cattle domestication, whereas newly evolving functions have been suggested for these genes based on their reported duplication in the cattle genome (Elsik et al., 2009). Regarding the large number of olfactory receptor genes included in the Multi-breed CSS regions, it should be taken into account that this gene family shows one of the highest frequency of somatic mutations in their coding regions due to low expression levels, late replication time during the cell cycle and high regional non-coding mutation rate (Lawrence et al., 2013). This observation may suggest these genes as false positive results in GWAS analyses, as pointed out by Lawrence et al. (2013), and may also be relevant in interpreting results from selection signature analyses.

As mentioned above, about 90% of the CSSs defined involved a single breed (57%) or a limited number of breeds (33%, Two-to-Four CSSs). The Single-breed CSSs included an over-representation of genes related to dairy and beef production; this observation was supported by the functional enrichment analysis, which highlighted production-related pathway terms associated with these regions (Table 3). Hence, the Single-breed CSS regions may include genes with small effects that influence quantitative traits of economic interest. This also suggests that similar selective pressures on different breeds, for example, for milk and meat production traits, can result in allele frequency changes in different genomic regions. This interpretation agrees with the hypothesis that many genes influence the complex traits under selection in cattle and that few of them show large phenotypic effects (Hayes et al., 2010). Alternatively, although within the same production category (dairy, beef, dual-purpose), the breeds may have been selected for subtly different production characteristics or have been subjected to differential natural (environmental) selection. In any case, each breed retains its own unique signature of its selection history. The functional enrichment analysis performed for the dual-purpose breeds, for which extremely strong selection has not been performed on either dairy or beef traits, primarily revealed genes related to reproduction traits and behavior-physiological pathways. Overall, the Single-breed CSSs pinpoint specific regions that appear to have been uniquely selected in the corresponding breeds. We propose these regions as potential markers of unique diversity and further studies focusing on the molecular basis of these selection sweeps are recommended. Furthermore, we acknowledge that a more comprehensive review also covering *Bos indicus* and African *Bos taurus* cattle would provide an enhanced overview of the impact of artificial and natural selection on the cattle genome. For example, for a selection sweep that appears to be related to short, slick hair coat (which in turn is associated with heat-stress tolerance) in tropical Senepol cattle (Flori et al., 2012), a mutation in the *PRLR* (prolactin receptor) has been identified as the putative causal mutation (Littlejohn et al., 2014). The identification of this effect, associated with a gene of major importance in lactation, provides a clear example of pleiotropy and the complex genetic architecture of physiological traits and suggests that examining selection sweeps in a broader range of cattle breeds could help to dissect the genetic architecture of traits of economic relevance.

This large-scale review of selection sweeps in European cattle reveals the historical impacts of long-term selection pressures on a species of great importance in human history. This review also presents for the first time a characterization of the selection sweeps that are breed-specific, and suggests that based on their uniqueness, these could be considered as “divergence signals,” which may be important for the management and prioritization of livestock diversity.

### Conflict of interest statement

The authors declare that the research was conducted in the absence of any commercial or financial relationships that could be construed as a potential conflict of interest.
